# Genetic Association of Objective Sleep Phenotypes with a Functional Polymorphism in the Neuropeptide S Receptor Gene

**DOI:** 10.1371/journal.pone.0098789

**Published:** 2014-06-04

**Authors:** Janek Spada, Christian Sander, Ralph Burkhardt, Madlen Häntzsch, Roland Mergl, Markus Scholz, Ulrich Hegerl, Tilman Hensch

**Affiliations:** 1 LIFE – Leipzig Research Center for Civilization Diseases, Universität Leipzig, Leipzig, Germany; 2 Department of Psychiatry and Psychotherapy, Universität Leipzig, Leipzig, Germany; 3 Institute of Laboratory Medicine, Clinical Chemistry and Molecular Diagnostics, University Hospital Leipzig, Leipzig, Germany; 4 Institute for Medical Informatics, Statistics and Epidemiology (IMISE), Universität Leipzig, Leipzig, Germany; University of Wuerzburg, Germany

## Abstract

**Background:**

The neuropeptide S receptor (NPSR1) and its ligand neuropeptide S (NPS) have received increased attention in the last few years, as both establish a previously unknown system of neuromodulation. Animal research studies have suggested that NPS may be involved in arousal/wakefulness and may also have a crucial role in sleep regulation. The single nucleotide polymorphism (SNP) rs324981 in *NPSR1* has begun to shed light on a function of the NPS-system in human sleep regulation. Due to an amino acid exchange, the T-allele leads to an increased sensitivity of the NPSR1. In the only genome-wide association study to date on circadian sleep parameters in humans, an association was found between rs324981 and regular bedtime. However, the sleep parameters in this study were only measured by self-rating. Therefore, our study aimed to replicate these findings using an objective measure of sleep.

**Methods:**

The study included n = 393 white subjects (62–79 years) who participated in an actigraphic assessment for determining sleep duration, rest duration, sleep onset, rest onset and sleep onset latency. Genotyping of the SNP rs324981 was performed using the TaqMan OpenArray System.

**Results:**

The genotype at rs324981 was not significantly associated with rest onset (bedtime) or sleep onset (p = .146 and p = .199, respectively). However, the SNP showed a significant effect on sleep- and rest duration (p = .007 and p = .003, respectively). Subjects that were homozygous for the minor T-allele had a significantly decreased sleep- and rest duration compared to A-allele carriers.

**Conclusion:**

The results of this study indicate that the sleep pattern in humans is influenced by the NPS-system. However, the previously reported association between bedtime and rs324981 could not be confirmed. The current finding of decreased sleep duration in T/T allele carriers is in accordance with studies in rodents reporting similar results after NPS application.

## Introduction

The neuropeptide S receptor (NPSR1) is a metabotropic G-protein coupled receptor with seven transmembrane helices [1]. The receptor was first described in 2002 and was deorphanized in 2004 by the identification of neuropeptide S (NPS) as its ligand [2], [3]. NPS belongs to the neuropeptides, a diverse group of neuronal expressed signalling molecules involved in a variety of brain functions. Studies of rats have demonstrated that the injection of NPS strongly induces wakefulness and reduces the occurrence of all sleep stages [4], [2].

NPS seems to be expressed in only a few defined regions (with possibly wide-ranging neuronal projections), which supports the notion of a neuromodulatory function of the NPS-system [5]. The highest concentration of NPS precursor mRNA has been found in brainstem neurons adjacent to the locus coeruleus, in the parabrachial nucleus and in the principle sensory trigeminal nucleus [2], [4]. Both the locus coeruleus area and the parabrachial nucleus are known for their contribution in the ascending arousal network, and the sensory trigeminal nucleus is also strongly modulated by the sleep/wake cycle [6], [7]. Compared to the NPS expression pattern, the NPSR1 precursor mRNA is distributed more widely in the brain. It covers important hubs of the sleep/arousal system in the hypothalamus and thalamus, but is also present in the cortex and the amygdala. In particular, it can be found in hypothalamic regions, like the perifornical region and the tuberomammilary nucleus, which are known for their expression of the wake-promoting orexin and histamine respectively [8]–[10]. Moreover, NPSR1 mRNA has been found in the key regions responsible for sleep induction.

On the molecular level, the receptor activates protein kinases and increases the intracellular cAMP and Ca^2+^ level [2]. In this way, NPS is thought to modulate the neurotransmission of the NPSR1 expressing neurons.

Although the NPS-system seems to play a crucial role in sleep modulation, most of the findings were derived from studies of rodents, and limited data is available on its effect on sleep in humans. The single nucleotide polymorphism rs324981 (lying at triplet position 107 of the *NPSR1* gene on chromosome 7p14.3), provides the opportunity to non-invasively study the effect of NPS/NPSR1 in humans. The T-allele of the SNP leads to an amino acid exchange in the active centre of the receptor binding site (Asn -> Ile). This causes approximately a tenfold increased sensitivity to neuropeptide S [11]. The T-allele has already been identified as a risk-factor for asthma and panic-disorder, the symptomatology of which might be partially driven or worsened by a pathologically altered arousal [12]–[14]. A genome wide association study (GWA) by Gottlieb et al. [15], which investigated the sleep-related parameter usual bedtime (the time a person goes to bed) and sleep duration, found an association between rs324981 and bedtime. This study was able to show a delay of usual bedtime in T-allele carriers. However, the sleep parameters in this study were only measured subjectively, through two single questionnaire items. Thus, our study aimed to replicate the influence of the functional *NPSR1* polymorphism on the sleep pattern via an objective measure of sleep. Based on the findings of Gottlieb et al. and the results from physiological studies, we hypothesized a delay in sleep/rest onset for T-allele carriers as well as a decreased sleep/rest duration.

## Methods

### Ethics Statement

All subjects gave written informed consent to participate in the study. The procedures were conducted according to the Declaration of Helsinki and approved by the University of Leipzig's ethics committee (registration-number: 263-2009-14122009).

### Subjects

Subjects were participants of the large-scale research project ‘LIFE’ (Leipzig Research Center for Civilisation Diseases). Within LIFE, a population based cohort of adult persons (40–79 years) was recruited in Leipzig (district), Germany. The subjects underwent a deep phenotyping, including blood sampling. A proportion of the elderly subjects (>60 years) also participated in an actigraphic assessment. All subjects were systematically screened for neurological conditions and medication use. Moreover, subjects were examined for psychiatric disorders using the structured clinical interview (SKID-I).

Actigraphic sleep data and rs324981 SNP genotype data were available in n = 436 elderly white subjects. Subjects with less than 5 days of actigraphic data were excluded from analysis (n = 14). Additional subjects were excluded due to neurological or psychiatric disorders or the use of psychotropic drugs (n = 29). In detail, exclusion criteria comprised current anxiety disorders (panic disorder, generalized anxiety disorder, posttraumatic stress disorder, obsessive–compulsive disorder, social phobia, specific phobia), current affective disorders (major depression, bipolar disorder, mania, dysthymia, cyclothymia), psychotic disorders (e.g. schizophrenia) and major neurological conditions (Parkinson's disease, multiple sclerosis, stroke and epilepsy). Exclusion criteria further comprised psychotropic drugs, such as antidepressants, neuroleptics, benzodiazepines and z-hypnotics. The remaining 393 subjects (females  = 175), aged between 62 and 79 years (mean: 70.5 years; SD: 3.6 years), were suitable for analysis.

### Genotyping

Genomic DNA was extracted from EDTA treated blood samples using the Autopure LS instrument (Qiagen, Hilden). Genotyping of SNP rs324981 was performed using TaqMan SNP Genotyping Assays on OpenArray-Chips (Applied Biosystems; Foster City, California). The call rate was 99.7% and the allele frequency was within the Hardy-Weinberg equilibrium (HWE; minor allele frequency  = 47%; pHWE  = 0.45). Genotyping was performed in the Institute of Laboratory Medicine, Universität Leipzig.

### Actigraphy

For objectively determining parameters of sleep, the activity of the subjects was measured using the SenseWear Pro 3 actigraph (BodyMedia; Pittsburgh, Pennsylvania). It was attached to the upper right arm, recording data about 2-axis body acceleration, skin temperature, heat flux and galvanic skin response. Based on these sensory parameters, the Sensewear algorithm identified periods of sleep or rest. Validation studies have shown that the Sensewear actigraph accurately detects sleep, as compared to the “gold standard“ polysomnography [16]. Subjects of the analysis sample wore the actigraph for an average of 6.3 days (range: 5–7 days), with the recording interval set to 1 minute. The wearing and removal of the actigraph was detected by an off-body detection. Days were defined as the 24 h interval from 12:00 pm (mid-day) to 11:59 am of the following day, thus covering the night sleep interval completely. Only days with a wearing time of at least 1200 min (20 h), and without gaps in the relevant night sleep time window were included in the study. For each subject, actigraphy raw data was processed in a separate file, based on a custom Excel template with Visual Basic for Applications (VBA) macros (Microsoft; Redmond), which computed all sleep variables automatically. To differentiate between night sleep and day sleep, the respective sleep intervals were tagged, referring to the time of day and a sleep diary that was kept by the subjects in parallel with the actigraphic assessment. The current study only focused on sleep that occurred at night time. The observed sleep variables comprised of the following: beginning of sleep (sleep onset), bedtime (rest onset), the time period subjects were at sleep (sleep duration), the time period subjects stayed in bed (rest duration) and the time it took subjects to fall asleep (sleep onset latency). Sleep onset latency was defined as the rest interval prior to sleep onset that was not interrupted by more than 1 min of activity. Rest onset was defined as the beginning of this rest interval.

### Statistical Analysis

Statistical analysis was performed with SPSS Statistics 21 (2012; IBM corp.; Armonk, New York). The sleep parameters used for analysis were calculated as the individual means across all nights matching the above criteria. Different genetic inheritance models were tested for their suitability to the data by using the Akaike information criterion (AIC) [17], [18]. Based on the lowest mean AIC score, the T-recessive model was identified as providing the best fit of all models (co-dominant or additive, dominant, recessive, and over-dominant). For testing the genetic association between the rs324981 genotype and sleep, a Multivariate Analysis of Covariance (MANCOVA) was conducted. The five sleep phenotypes mentioned above served as multivariate endpoints of our analyses. Sex, age and body mass index (BMI) were included as covariates, as these factors have shown to affect the sleep pattern [19], [20]. Univariate follow-up tests were conducted separately for each sleep parameter. In all univariate analyses, the alpha-level was corrected for multiple testing by using Bonferroni’s method. All statistical tests were two-sided. The sleep variables were normally distributed (Kolmogorov-Smirnov-Test; p>0.05), except for sleep onset latency which had a lognormal-like distribution [21], [22]. Therefore, an inverse hyperbolic sine (IHS) transformation was applied on sleep onset latency before statistical analysis. The IHS transformation is similar to a log transformation, but defined at zero [23]. For improving the interpretability, mean values and confidence intervals (CI) were back-transformed after analysis.

## Results

We first investigated the influence of covariates on the sleep parameters. We observed significant effects for sex (p = .009) and BMI (p<.001) on sleep duration (with shorter duration in men and in subjects with higher BMI). BMI was furthermore associated with rest duration (p = .001), sleep onset (p = .002), rest onset (p = .015) and sleep onset latency (p<.001).

Multivariate analysis showed a significant association between the *NPSR1* genotype at rs324981 and sleep, as assessed with all five sleep-related parameters (F_(5, 384)_ = 2.262, p = .048, η_p_^2^ = .029). This effect was mainly due to the significant impact on sleep duration (F_(1, 388)_ = 7.400, p = .007, η_p_^2^ = .019) and rest duration (F_(1, 388)_ = 8.853, p = .003, η_p_^2^ = .022). Sleep- as well as rest- duration were shorter in individuals with the homozygote T/T genotype, than in A-allele carriers. When separated for sex the same trend was seen in both females and males. In accordance with this, we found no significant interaction between sex and genotype (sex x genotype: sleep duration, F_(1, 387)_ = .198, p = .656; rest duration, F_(1, 387)_ = .028, p = .868).

Sleep onset, rest onset, and sleep onset latency were not significantly associated with the SNP genotype at rs324981 (F_(1, 388)_ = 1.654, p = .199, η_p_^2^ = .004, F_(1, 388)_ = 2.127, p = .146, η_p_^2^ = .005 and F_(1, 388)_ = 1.698, p = .193, η_p_^2^ = .004, respectively; Table 1). None of these effects interacted with sex, age or BMI.

**Table 1:** Association results between the *NPSR1* SNP (rs324981) genotype and actigraphic sleep parameters.

phenotype	genotype				p-value
	A/A+A/T [n = 120+199]		T/T [n = 74]		
	mean	95% CI	mean	95% CI	
sleep duration [h:m]	6∶31	{6∶25, 6∶38}	6∶11	{5∶57, 6∶24}	0.007*
rest duration [h:m]	7∶53	{7∶47, 7∶59}	7∶33	{7∶21, 7∶45}	0.003*
sleep onset latency^b^ [m]	7.94	{7.30, 8.64}	6.97	{5.84, 8.32}	0.193
sleep onset [h:m]	23∶18	{23∶12, 23∶23}	23∶26	{23∶15, 23∶37}	0.199
rest onset [h:m]	23∶07	{23∶02, 23∶13}	23∶16	{23∶05, 23∶28}	0.146

The table shows the covariate-adjusted means, 95% confidence intervals (CI) and p-values based on univariate analysis of covariance (ANCOVA; covariates: sex, age, BMI).

Footnotes: CI = confidence interval; h = hour; m = minute;

*significant at the corrected alpha level,

b back-transformed values (inverse hyperbolic sine transformation).

## Discussion

The current study illustrates evidence for an association between *NPSR1* and objectively obtained sleep parameters in humans. The functional SNP rs324981, located in the gene encoding NPSR1, was found to have a significant effect on sleep duration and rest duration in a sample of elderly subjects. Subjects with the homozygous T/T genotype had a significantly shorter sleep- and rest duration compared to subjects carrying the A-allele. These findings partially confirm the GWA study by Gottlieb et al. [15] in terms of a general association between rs324981 and sleep-related traits in humans. However, the current study was not able to replicate an effect of the rs324981 genotype upon rest onset (bedtime). Even though we also observed a later rest onset in homozygous T-allele carriers in the current sample, this effect failed to reach statistical significance.

A possible explanation for this discrepancy may be accounted for by the difference between the methodical approaches. Measuring sleep by actigraphy is far more objective than the traditional assessment by questionnaire. In fact, there is only a moderate correlation between self-reported and objectively measured sleep duration [24]. Subjective assessment is consistently found to be an overestimation of sleep duration and sleep onset [25], [26]. Moreover, the study by Gottlieb et al. [15] used only a single question asking for usual bedtime/sleep duration, and did not collect data about multiple nights. The higher mean age of the current sample (Δ 14.7 years) might also partially explain the differing results. Most of the subjects were in retirement age, which is advantageous in that job engagement is not inhibiting or influencing sleep preferences. However, since circadian sleep habits change with increasing age [27], the respective genetic associations might be less pronounced in the elderly. In the age range of the current sample we observed no influence of age on any sleep parameter. Although the present study provides a more objective measure of bedtime, the sample size was smaller than in the study by Gottlieb et al. (n = 393 vs. n = 738). Therefore we cannot rule out that the association was too weak to be detected with the available statistical power.

Our finding of an association between rs324981 and sleep duration is consistent with studies in rats, reporting that direct application of NPS into the brain, strongly influences the sleep architecture. In the study by Xu et al. [2], rats spent significantly more time in wakefulness, compared to rapid eye movement sleep (REM), as well as slow wave sleep (SWS) phases I and II, which were shortened during the first hour after NPS application. Similar results were found by Zhao et al. [4], who reported decreased sleep phases and increased high frequency power in the sleep EEG. These studies indicate that NPS can inhibit both REM and non-REM sleep phases, which are thought to be regulated by partially independent systems [28]. Since the T-allele increases the sensitivity of NPSR1 towards NPS, the current finding of a shorter mean sleep duration in T/T carriers, is in line with the expectations. The causal relationship however, might be far more complicated since it is not known how the rs324981 polymorphism acts during ontogenesis. It has been hypothesised that gain-/loss-of-function alleles may induce compensatory mechanisms or interact with other unknown genetic/environmental factors [29]–[31].

The functional mechanism underlying the effect of NPS/NPSR1 on sleep and arousal is not well understood. Generally, sleep and wakefulness are thought to be regulated by at least two antagonizing brain systems; the arousal- and the sleep-promoting system. The arousal system mainly originates in the brainstem and in the lateral hypothalamus, innervating the forebrain and the cortex, where the system is thought to modulate cortical activity [32]. Strong NPSR1 mRNA expression was detected in the lateral hypothalamus, including orexinergic neurons in the perifornical region as well as the histaminergic tuberomammilary nucleus [9]. A recent study was able to show that NPS application enhances the hypothalamic expression of c-Fos (an indirect marker of neuronal activity) in the respective histaminergic and orexinergic regions [4]. It was therefore proposed that the arousal-promoting effect of NPS is partially mediated by the release of histamine and orexin. Histaminergic neurons have widespread projections and normally show high activity during wakefulness, and are inactive during sleep [28]. Pharmacological interventions with histamine receptor agonists/antagonists have repeatedly demonstrated the influence of histamine on the wake/sleep cycle [33]. Orexin is, similar to NPS, a neuropeptide with a wake-promoting effect [8]. Loss of the orexinergic neurons is known to be a cause of narcolepsy [34]. NPSR1 mRNA is also present in brain regions, which are assigned to the sleep-promoting system; the lateral preoptic area, the ventrolateral preoptic nucleus and the nucleus of the horizontal limb of the diagonal band [9]. Whether NPSR1 has an inhibiting effect in these neurons remains to be investigated. In vivo, NPS might be co-released from the brainstem neurons of the ascending arousal sytem (i.e. potentially from neurons near the locus coeruleus and the parabrachial nucleus) [9], [4]. Immunohistochemical analyses indicate that NPS-immunopositive neurons directly project to NPSR1 expressing neurons in the hypothalamus [35]. In this way, NPS might modify the effect of the regular neurotransmitters on arousal-promoting or sleep-inhibiting neurons [9].

The findings of the current study might have positive implications for the continuing research into the molecular genetics of sleep. This is not only important for developing new treatments for sleep problems, but also for understanding the comorbidity of sleep and psychiatric conditions, as sleep and arousal are strongly linked to the pathophysiology and treatment of disorders such as affective disorders and attention deficit hyperactivity disorder [36]–[39].

## Conclusions

The functional SNP rs324981 located in the gene of NPSR1 was significantly associated with objectively obtained sleep parameters in a sample of elderly white subjects. Although, the previous finding of an effect on self-reported bedtime could not be confirmed by using actigraphy, the findings still point to a role of the NPS system in human sleep. As sleep has a high association with well-being, cognitive abilities as well as psychological and physical health, this study emphasizes the need for more research to determine the specific function of NPS/NPSR1 in the human brain.

## References

[1] Pitti T, Manoj N, Uversky VN (2012) Molecular Evolution of the Neuropeptide S Receptor. PLoS ONE 7 (3): e34046. 22479518 10.1371/journal.pone.0034046 PMC3316597

[2] Xu Y, Reinscheid RK, Huitron-Resendiz S, Clark SD, Wang Z, et al (2004) Neuropeptide S. Neuron 43 (4): 487–497. 15312648 10.1016/j.neuron.2004.08.005

[3] Sato S, Shintani Y, Miyajima N, Yoshimura K (2002) Novel G protein-coupled receptor protein and DNA thereof. World Patent Application. WO 02/31145 A1.

[4] Zhao P, Shao YF, Zhang M, Fan K, Kong XP, et al (2012) Neuropeptide S promotes wakefulness through activation of the posterior hypothalamic histaminergic and orexinergic neurons. Neuroscience 207: 218–226. 22300983 10.1016/j.neuroscience.2012.01.022

[5] Reinscheid R (2008) Neuropeptide S: Anatomy, Pharmacology, Genetics and Physiological Functions. In: Civelli O, Zhou Q, editors. Orphan G Protein-Coupled Receptors and Novel Neuropeptides: Springer Berlin Heidelberg. pp. 145–158.

[6] Cairns BE, Fragoso MC, Soja PJ (1995) Activity of rostral trigeminal sensory neurons in the cat during wakefulness and sleep. J Neurophysiol 73 (6): 2486–2498. 7666154 10.1152/jn.1995.73.6.2486

[7] Kohlmeier KA, Soja PJ, Kristensen MP (2006) Disparate cholinergic currents in rat principal trigeminal sensory nucleus neurons mediated by M1 and M2 receptors: a possible mechanism for selective gating of afferent sensory neurotransmission. Eur J Neurosci 23 (12): 3245–3258. 16820015 10.1111/j.1460-9568.2006.04875.x

[8] Sutcliffe JG, De Lecea L (2002) The hypocretins: setting the arousal threshold. Nat Rev Neurosci 3 (5): 339–349. 11988773 10.1038/nrn808

[9] Xu Y, Gall CM, Jackson VR, Civelli O, Reinscheid RK (2007) Distribution of neuropeptide S receptor mRNA and neurochemical characteristics of neuropeptide S-expressing neurons in the rat brain. J Comp Neurol 500 (1): 84–102. 17099900 10.1002/cne.21159

[10] Jones BE (2003) Arousal systems. Front Biosci 8: s438–51. 12700104 10.2741/1074

[11] Reinscheid RK (2005) Pharmacological Characterization of Human and Murine Neuropeptide S Receptor Variants. J Pharmacol Exp Ther 315 (3): 1338–1345. 16144971 10.1124/jpet.105.093427

[12] Domschke K, Reif A, Weber H, Richter J, Hohoff C, et al (2010) Neuropeptide S receptor gene — converging evidence for a role in panic disorder. Mol Psychiatry 16 (9): 938–948. 20603625 10.1038/mp.2010.81

[13] Glotzbach-Schoon E, Andreatta M, Reif A, Ewald H, Troger C, et al (2013) Contextual fear conditioning in virtual reality is affected by 5HTTLPR and NPSR1 polymorphisms: effects on fear-potentiated startle. Front Behav Neurosci 7: 31. 23630477 10.3389/fnbeh.2013.00031 PMC3632789

[14] Laas K, Reif A, Akkermann K, Kiive E, Domschke K, et al (2014) Interaction of the neuropeptide S receptor gene Asn107Ile variant and environment: contribution to affective and anxiety disorders, and suicidal behaviour. Int J Neuropsychopharmacol 17(4): 541–552. 24331455 10.1017/S1461145713001478

[15] Gottlieb DJ, O'Connor GT, Wilk JB (2007) Genome-wide association of sleep and circadian phenotypes. BMC Med Genet 8 Suppl 1 S9. 17903308 10.1186/1471-2350-8-S1-S9 PMC1995620

[16] Sharif MM, Bahammam AS (2013) Sleep estimation using BodyMedia's SenseWear armband in patients with obstructive sleep apnea. Ann Thorac Med 8 (1): 53–57. 23440703 10.4103/1817-1737.105720 PMC3573559

[17] Akaike H (1974) A new look at the statistical model identification. IEEE Trans Automat Contr 19 (6): 716–723.

[18] Minelli C, Thompson JR, Abrams KR, Thakkinstian A, Attia J (2005) The choice of a genetic model in the meta-analysis of molecular association studies. Int J Epidemiol 34 (6): 1319–1328. 16115824 10.1093/ije/dyi169

[19] Krishnan V, Collop NA (2006) Gender differences in sleep disorders. Curr Opin Pulm Med 12 (6): 383–389. 17053485 10.1097/01.mcp.0000245705.69440.6a

[20] Moraes W, Poyares D, Zalcman I, De Mello MT, Bittencourt LR, et al (2013) Association between body mass index and sleep duration assessed by objective methods in a representative sample of the adult population. Sleep Med 14 (4): 312–318. 23391395 10.1016/j.sleep.2012.11.010

[21] Von Arb M, Flammer J, Gompper B, Kräuchi K, Meyer AH, et al (2009) Relationship between gender role, anger expression, thermal discomfort and sleep onset latency in women. BioPsychoSocial Med 3 (1): 11. 10.1186/1751-0759-3-11 PMC2770539 19825177

[22] Raymann RJ, Swaab DF, Someren EJ van (2007) Skin temperature and sleep-onset latency: Changes with age and insomnia. Physiol Behav 90 (2–3): 257–266. 17070562 10.1016/j.physbeh.2006.09.008

[23] Burbidge JB, Magee L, Robb AL (1988) Alternative Transformations to Handle Extreme Values of the Dependent Variable. J Am Statist Assoc 83 (401): 123–127.

[24] Lauderdale DS, Knutson KL, Yan LL, Liu K, Rathouz PJ (2008) Self-Reported and Measured Sleep Duration. Epidemiology 19 (6): 838–845. 18854708 10.1097/EDE.0b013e318187a7b0 PMC2785092

[25] Wang M, Hung H, Tsai P (2011) The Sleep Log and Actigraphy. J Nurs Res 19(3): 173–180. 21857324 10.1097/JNR.0b013e318229c42f

[26] Lockeley SW, Skene DJ, Arendt J (1999) Comparison between subjective and actigraphic measurement of sleep and sleep rhythms. J Sleep Res 8(3): 175–183. 10476003 10.1046/j.1365-2869.1999.00155.x

[27] Roenneberg T, Kuehnle T, Juda M, Kantermann T, Allebrandt K, et al (2007) Epidemiology of the human circadian clock. Sleep Med Rev 11 (6): 429–438. 17936039 10.1016/j.smrv.2007.07.005

[28] Brown RE, Basheer R, McKenna JT, Strecker RE, McCarley RW (2012) Control of Sleep and Wakefulness. Physiol Rev 92 (3): 1087–1187. 22811426 10.1152/physrev.00032.2011 PMC3621793

[29] Klauke B, Deckert J, Zwanzger P, Baumann C, Arolt V, et al (2012) Neuropeptide S receptor gene (NPSR) and life events: G×E effects on anxiety sensitivity and its subdimensions. World J Biol Psychiatry 15 (1): 17–25. 22404660 10.3109/15622975.2011.646302

[30] Hunter DJ (2005) Gene-environment interactions in human diseases. Nat Rev Genet 6 (4): 287–298. 15803198 10.1038/nrg1578

[31] Gibson G (2009) Decanalization and the origin of complex disease. Nat Rev Genet 10 (2): 134–140. 19119265 10.1038/nrg2502

[32] Saper CB, Scammell TE, Lu J (2005) Hypothalamic regulation of sleep and circadian rhythms. Nature 437 (7063): 1257–1263. 16251950 10.1038/nature04284

[33] Thakkar MM (2011) Histamine in the regulation of wakefulness. Sleep Med Rev 15 (1): 65–74. 20851648 10.1016/j.smrv.2010.06.004 PMC3016451

[34] De la Herrán-Arita AK, Guerra-Crespo M, Drucker-Colín R (2011) Narcolepsy and Orexins: An Example of Progress in Sleep Research. Front Neur 2: 26. 10.3389/fneur.2011.00026 PMC3082766 21541306

[35] Clark SD, Duangdao DM, Schulz S, Zhang L, Liu X, et al (2011) Anatomical characterization of the neuropeptide S system in the mouse brain by in situ hybridization and immunohistochemistry. J Comp Neurol 519 (10): 1867–1893. 21452235 10.1002/cne.22606

[36] Hegerl U, Wilk K, Olbrich S, Schoenknecht P, Sander C (2012) Hyperstable regulation of vigilance in patients with major depressive disorder. World J Biol Psychiatry 13 (6): 436–446. 21722018 10.3109/15622975.2011.579164

[37] Hegerl U, Hensch T (2012) The vigilance regulation model of affective disorders and ADHD. Neurosci Biobehav Rev: In press. 10.1016/j.neubiorev.2012.10.008 23092655

[38] Sander C, Arns M, Olbrich S, Hegerl U (2010) EEG-vigilance and response to stimulants in paediatric patients with attention deficit/hyperactivity disorder. Clin Neurophysiol 121 (9): 1511–1518. 20382071 10.1016/j.clinph.2010.03.021

[39] Hegerl U, Himmerich H, Engmann B, Hensch T (2010) Mania and attention-deficit/hyperactivity disorder: common symptomatology, common pathophysiology and common treatment. Curr Opin Psychiatry 23 (1): 1–7. 19770771 10.1097/YCO.0b013e328331f694

